# The Relationship Between Problematic Internet Use (PIU) and Psychological Distress: A cross- sectional study

**DOI:** 10.1192/j.eurpsy.2024.832

**Published:** 2024-08-27

**Authors:** M. Theodoratou, M. Varvitsioti, G. A. Kougioumtzis, G. Tsitsas, A. Kaltsouda, K. Flora, C. Papathanasiou

**Affiliations:** ^1^Social Sciences, Hellenic Open University, Patras, Greece; ^2^Health Sciences, Neapolis University Pafos, Pafos, Cyprus; ^3^Psychology, National and Kapodistrian University; ^4^Psychology, Harokopion University, Athens; ^5^Psychology, University of Ioannina, Ioannina; ^6^Psychology, Macedonian University, Florina, Greece

## Abstract

**Introduction:**

Problematic Internet Use (PIU) has emerged as a widespread social challenge and is characterised by an individual’s inability to regulate their internet use, culminating in a negative impact on their daily life. It is vital to explore the possible mediating relationship between psychological distress and unregulated Internet use.

**Objectives:**

The study aims to explore the relationship between problematic Internet use, psychological distress and quality of life.

**Methods:**

In this cross-sectional study, participants engaged in a structured data collection process using Google Forms, responding to a series of questions developed through a quantitative methodology using a Likert scale questionnaire.

The K-6 Distress Scale and the World Health Organisation Quality of Life (WHOQOL) were used in conjunction with questions about demographics and problematic internet use.The integration of these multiple measures aimed to provide comprehensive insights into the effects and patterns of Internet use and its association with different levels of distress and demographic variables.

**Results:**

Statistical data analysis revealed notable associations between psychological distress and several variables, including age, education, employment status and health, but no significant associations were found with place of residence or marital status. Significant associations were also found between problematic Internet use (PIU) and variables such as age, education, employment, marital status and health. However, no significant association was found with place of residence. Quality of life was also found to be correlated with age, employment status and health, but not with marital status or educational attainment. In addition, the analysis revealed a strong association between problematic internet use (PIU) and psychological distress. A concurrent increase in reported psychological distress was observed as PIU levels escalated, reinforcing the relationship between excessive internet use and psychological wellbeing. This suggests that PIU may be associated with increased levels of mental distress in the population studied.

**Conclusions:**

In conclusion, this study provides valuable insights into the multifaceted dynamics of mental distress, PIU, and quality of life in relation to demographic factors. It underscores the importance of holistic approaches to mental health that consider individual characteristics and behaviors, with a particular focus on addressing the challenges posed by problematic internet use. Further research and tailored interventions are needed to better understand and support people facing these complex issues.

**Disclosure of Interest:**

None Declared

